# Coronavirus disease 2019 related parosmia: an exploratory survey of demographics and treatment strategies

**DOI:** 10.1017/S0022215123000713

**Published:** 2023-11

**Authors:** L J Sowerby, Z Almubarak, A Biadsee, T Rocha, C Hopkins

**Affiliations:** 1 Department of Otolaryngology – Head and Neck Surgery, Schulich School of Medicine and Dentistry, Western University, London, Ontario, Canada; 2 Department of Otorhinolaryngology – Head and Neck Surgery, Meir Medical Center, Kfar-Saba, Israel; 3 Sackler Faculty of Medicine, Tel Aviv University, Tel Aviv, Israel; 4 Department of ENT, Guy's Hospital, London, UK

**Keywords:** Olfaction disorders, post-acute COVID-19 syndrome, smell, self-help groups, SARS-CoV-2, olfactory training

## Abstract

**Objective:**

To investigate the clinical features, therapeutic efficacy and symptom time course of post-coronavirus disease 2019 parosmia.

**Methods:**

A 22-item online questionnaire was distributed to AbScent research group and Facebook coronavirus disease 2019 anosmia group adult members to assess clinical features, interventions and their subjective efficacy for parosmia.

**Results:**

A total of 209 participants (86 per cent females) reported: smell loss on average 3 days after coronavirus symptoms, recovery 4 weeks later, and first parosmia symptoms 12 weeks post infection. Respondents reported 10 per cent body weight loss, and listed onion and garlic as significant parosmia triggers. Regarding quality of life, depression was the most cited item (54 per cent). Smell training was trialled by 74 per cent of participants, followed by nasal corticosteroid spray (49 per cent). Stellate ganglion block, trialled by 16 per cent of respondents, had the highest reported improvement (45 per cent), with 21 per cent reporting a sustained benefit – the highest rate amongst registered treatment options.

**Conclusion:**

Post-coronavirus parosmia has a significant impact and remains challenging to treat. Stellate ganglion block appears to be successful relative to other reported treatments. Further research into the pathophysiology, efficacy and mechanism of stellate ganglion block effect is warranted.

## Introduction

Infection with severe acute respiratory syndrome coronavirus 2 (SARS-CoV-2) was declared a pandemic in March 2020 by the World Health Organization. Since then, more than six million deaths have been registered due to coronavirus disease 2019 (Covid-19).^1^ Clinical manifestations vary between patients; typically, patients present with respiratory symptoms such as coughing, shortness of breath, sore throat or fever.^2^ A more severe form is predominant in older patients or patients with chronic conditions, causing acute respiratory distress syndrome and multi-organ failure.^2–6^

From the pandemic's early days, olfactory dysfunction (subjective and objective) has been described in many patients infected with SARS-CoV-2. Often olfactory dysfunction is the earliest or the only symptom.^7^ The prevalence of quantitative olfactory dysfunction ranges from 33 to 68 per cent among symptomatic Covid-19 patients across studies.^8,9^ Among the types of olfactory dysfunction, post-Covid-19 parosmia has been reported with an incidence ranging from 8 to 32 per cent during the disease course.^7,10^ The prevalence of quantitative olfactory dysfunction ranges from 33 to 68 per cent among symptomatic Covid-19 patients across studies.^8,9^ Existing literature on the symptomatology, time course and quality of life among those with parosmia is sparse, despite the significant morbidity it carries for some.^1^

Parosmia is defined as a distorted sense of smell, and has been reported in Covid-19 patients with a possible delayed presentation, yet its pathophysiology is still unclear.^11,12^ Parosmia has been suggested to be a consequence of deficits within integrative centres in the brain or the peripheral pathways abnormally transmitting odour stimulation.^12^ Although olfactory dysfunction has been described frequently in published data during the Covid-19 pandemic, minimal data have been published on post-Covid-19 parosmia.^2^

Different treatment modalities have been proposed, including corticosteroids, stellate ganglion block and olfactory training.^13–15^ Furthermore, vaccination has been suggested not only as a protective modality of severe disease but also as a potential intervention to reduce the sequelae of Covid-19 infection, one of which is the presentation or persistence of parosmia.^13^ The present study aimed to assess clinical features associated with developing parosmia among post-Covid-19 patients with olfactory dysfunction, the different interventions trialled in treating post-Covid-19 parosmia and their subjective efficacy in the resolution of symptoms.

## Materials and methods

This virtual cross-sectional survey study targeting adult patients with post-Covid-19 parosmia was approved by the Western University Institutional review board in London, Ontario, Canada (research ethics board number: 121512). Participants were invited through the AbScent research group (AbScent.org) consisting of 1000 patients and a Facebook (Meta, Menlo Park, California, USA) ‘Covid Anosmia/Parosmia Support Group’ (https://www.facebook.com/groups/232669991396703/) consisting of 50 000 members from 22 September to 22 October 2022.

A 22-item questionnaire with multiple-choice and open-ended questions was developed based on a critical review of previous literature, clinical expertise and reported therapies in patient forums (Supplement A). Informed consent was obtained virtually from participants, who could close and cancel participation in the survey at any time. The survey included questions on participants’ demographics, clinical and olfactory symptoms, vaccination status, temporal relationship to parosmia, and trialled treatments. The questionnaire was distributed via a REDCap (Research Electronic Data Capture) link to the AbScent research group and Facebook ‘Covid Anosmia/Parosmia Support Group’. Respondents aged younger than 18 years, and patients who reported not having experienced any smell or taste disturbance during or after Covid-19 infection, were excluded. Survey reminders were sent two and four weeks after the initial survey distribution. Final data were extracted two weeks after the second reminder.

Responses to the question ‘Do you know the strain of SARS-CoV-2 you had?’ that were marked ‘unknown’ were converted to Covid-19 variants based on the Centers for Disease Control and Prevention official timeline for Covid-19 variants prevalence. Infections in December 2020 and January 2021 were coded as Alpha variant. Infections occurring from February 2021 to July 2021 were coded as Beta variant. Infections from August 2021 to November 2021 were coded as Delta variant. Infections from December 2021 and later were coded as Omicron variant.^16^

Statistical analysis was performed using SPSS® version 26 software. An *a priori* alpha level of *p* < 0.05 was used to determine statistical significance. Survey respondents’ demographics and survey responses were reported descriptively using frequency, means and standard deviations (SDs), in addition to median and interquartile ranges where appropriate. Mean comparisons were performed using the independent samples *t*-test. Our study results are described per survey study reporting guidelines using the Checklist for Reporting of Survey Studies (‘CROSS’).^17^

## Results

The survey link was available for 30 days between September 2022 and October 2022, resulting in 312 accesses, with 214 complete survey responses (68 per cent). From these, one record was excluded because the reported age was less than 18 years old. Four records were excluded for reporting not having experienced any smell or taste disturbance during or after Covid-19 infection, resulting in a sample of 209 complete survey responses for analysis.

### Vaccination status pre-infection

Most respondents were from the USA (82 per cent), followed by the UK (8 per cent), Canada (3 per cent) and 11 other countries (up to 2 respondents each). On average, participants were aged 40 years (SD = 12, range = 18–68 years), with mostly female participants (86 per cent). Table 1 shows a summary of respondents' characteristics. Of the respondents, 104 (50 per cent) declared having received at least one dose of a Covid-19 vaccine, while 92 (44 per cent) had a second dose, 49 (23 per cent) had a third dose and 12 (6 per cent) had a fourth Covid-19 vaccine dose. See Table 2 for descriptions of vaccine types for each of these groups.

**Table 1.** Participants’ characteristics

Characteristics	Value
Age (mean (SD); years)	40 (12)
Sex (*n* (%))	
– Male	29 (14)
– Female	179 (85.5)
– Not reported	1 (0.5)
Country (*n* (%))	
– USA	170 (82)
– UK	17 (8)
– Canada	7 (3)
– Mexico	1 (0.5)
– Belgium	1 (0.5)
– Croatia	1 (0.5)
– Romania	1 (0.5)
– Australia	1 (0.5)
– South Africa	2 (0.9)
– Egypt	1 (0.5)
– Germany	2 (0.9)
– Finland	1 (0.5)
– Philippines	1 (0.5)
– North Macedonia	1 (0.5)
– Not reported	2 (1)
Vaccination status when infected with Covid-19 for 1st time (*n* (%))*	
– Not vaccinated	154 (80)
– Vaccinated	39 (20)
Covid-19 strain (*n* (%))	
– Omicron	73 (35)
– Delta	67 (32)
– Alpha	32 (15)
– Wild type (Wuhan)	25 (12)
– Beta	12 (6)

* *n* = 193; 16 participants did not report their first coronavirus disease 2019 (Covid-19) vaccine dose date, preventing assessment of their vaccination status at the time of infection. SD = standard deviation

**Table 2.** Brands of Covid-19 vaccines received by participants

Dose	Vaccine brand	*n* (%)
1st dose	Pfizer-BioNTech Comirnaty	64 (59)
	Moderna Spikevax	25 (23)
	Janssen (Johnson & Johnson)	11 (10)
	AstraZeneca Vaxzevria	8 (8)
2nd dose	Pfizer-BioNTech Comirnaty	96 (100)
3rd dose	Pfizer-BioNTech Comirnaty	26 (50)
	Moderna Spikevax	25 (48)
	Janssen (Johnson & Johnson)	1 (2)
4th dose	Pfizer-BioNTech Comirnaty	8 (61)
	Moderna Spikevax	5 (39)

Covid-19 = coronavirus disease 2019

Despite the vaccination rate described above, 154 respondents (80 per cent) were not vaccinated at the time of Covid-19 infection. Vaccinated respondents developed parosmia 88 days after infection, while unvaccinated respondents developed parosmia 124 days after infection, with a mean difference of 35.9 days (*p* = 0.019).

### Coronavirus variants and timing of symptoms

Initially, 60 per cent of participants responded ‘unknown’ to the Covid-19 variant question, followed by Delta (25 per cent), Alpha (6 per cent), wild type (5 per cent) and Omicron (3 per cent). No respondents reported the Beta variant, and one participant did not respond to this question. After converting the ‘unknowns’ and missing responses into Covid-19 variants based on the official Covid-19 variants prevalence timeline, the most prevalent variants among respondents (Table 1) were Omicron (35 per cent) and Delta (32 per cent), followed by Alpha (15 per cent), Wild type (12 per cent) and Beta (6 per cent).

Timing of symptoms related to loss of smell and taste during or after the Covid-19 infection showed heterogeneous data. Overall, study participants experienced smell and taste loss 3 days (median) (interquartile range, 2 days) after the first Covid-19 symptoms. Olfactory recovery was reported 4 weeks later (median) (interquartile range, 10 weeks), followed by the start of parosmia symptoms around 12 weeks after Covid-19 infection (median) (interquartile range, 15 weeks). The mean interval from infection to completion of the questionnaire was 67 weeks (SD = 29 weeks).

### Parosmia status

Eighty-seven respondents (42 per cent) declared having no improvement in their parosmia, while 45 participants (22 per cent) reported a 25 per cent improvement (interval from infection to completion of the questionnaire ranged from 28 to 135 weeks, mean = 71 weeks, SD = 27). Improvements of 50 per cent were reported by 34 respondents (16 per cent) and improvements of 75 per cent were reported by 33 respondents (16 per cent). The interval from infection to questionnaire completion for those who reported a 50 per cent improvement ranged from 43 to 146 weeks (mean = 77 weeks, SD = 27), while the interval for those who reported 75 per cent improvement ranged from 18 to 145 weeks (mean = 79 weeks, SD = 29). Full recovery (100 per cent improvement) was reported by only seven respondents (3 per cent); for these individuals, the interval from infection to questionnaire completion ranged from 6 to 100 weeks (mean = 72 weeks, SD = 34). Three participants did not respond to this question.

### Clinical implications and symptomatology

Weight loss due to post-Covid-19 parosmia was reported by 108 respondents (52 per cent), with a median 10 per cent loss of body weight (interquartile range, 11 per cent). On a scale of 1 to 10, respondents reported an average of 6 (SD = 2.5) in the severity of their parosmia symptoms. Sixty-seven participants (32 per cent) reported having a second Covid-19 infection. Of these 67 participants, when questioned whether their parosmia had changed after a second Covid-19 illness, 68 per cent answered that it was the same, 29 per cent said it was worse, and 3 per cent stated that their condition was better. The estimated expense incurred from post-Covid-19 parosmia treatments varied considerably within responses, ranging from $0 to $10 000 (CAD, 5800GBP) (median, $100CAD (58GBP); interquartile range median, $100.00; interquartile range, $679.00 (390GBP)).

From 191 responses to the open-ended question ‘What are the worst triggers for your parosmia?’, the 10 most often cited items were: onion (66 per cent), garlic (54 per cent), meat (48 per cent), coffee (40 per cent), chicken (25 per cent), eggs (16 per cent), peanut butter (15 per cent), chocolate (15 per cent), fried foods (13 per cent) and perfume (11 per cent). When 201 respondents answered the open-ended question, ‘How has parosmia affected your quality of life?’, depression was the most cited item (54 per cent), followed by appetite loss (40 per cent), weight loss (35 per cent), anxiety (22 per cent), weight gain (11 per cent), nausea (11 per cent), frustration (4 per cent), reduced energy (3 per cent) and mood swings (3 per cent). Among the less frequent items, it is worth noting that ‘suicidal thoughts’ was mentioned by 2.5 per cent of the respondents. Two participants reported needing hospitalisation: one due to Covid-19 infection complications and the other due to a suicide attempt.

### Treatment strategies

Most study participants (61 per cent) had seen their primary care provider regarding their parosmia, while only 27 per cent had sought help from an ENT specialist. Seven per cent of all respondents declared having tried each treatment option mentioned in the study survey. The most commonly used treatment amongst all respondents was smell training (74 per cent), followed by nasal corticosteroid spray (49 per cent) and vitamin A drops (20 per cent). See Table 3 for detailed descriptions of the trialled treatments.

**Table 3.** Trialled treatments for post-Covid-19 parosmia; success rates, treatment duration and additional details

Parameter	Smell training	Nasal steroid spray	Vitamin A drops	Nasal steroids irrigation	Oral steroids	Stellate ganglion block	Lion's Mane	Colloidal silver spray	Sodium citrate spray
Respondents who tried treatment (*n* (%))	155 (74)	103 (49)	42 (20)	40 (19)	40 (19)	33 (16)	34 (16)	14 (7)	13 (6)
Respondents who reported improvement (*n* (%))	15 (10)	3 (3)	2 (5)	0 (0)	5 (12)	15 (45)	3 (9)	0 (0)	0 (0)
Number of days until improvement (mean (SD))	49 (31)	6 (1)	30 (0)	n/a	10 (12)	n/a	43 (30)	n/a	n/a
Treatment duration (*n* (%))									
– 1 month	64 (41)	67 (69)	23 (55)	23 (65)	35 (92)	n/a	15 (47)	9 (64)	9 (75)
– 1–3 months	44 (29)	14 (15)	14 (33)	10 (28)	2 (5)	n/a	14 (44)	3 (21)	3 (25)
– >3 months	45 (29)	17 (17)	5 (12)	2 (7)	1 (3)	n/a	3 (9)	2 (15)	0 (0)
Respondents who reported maintained benefit (*n* (%))	9 (6)	3 (3)	2 (5)	n/a	2 (5)	7 (21)	3 (9)	n/a	n/a

Covid-19 = coronavirus disease 2019; SD = standard deviation; n/a = not applicable

Despite the lower percentage of participants who underwent a stellate ganglion block (16 per cent), this treatment presented the highest percentage of reported improvement (45 per cent), followed by oral steroids (12 per cent) and smell training (10 per cent). Of the respondents who reported improvement with stellate ganglion block, seven (21 per cent) reported a maintained benefit, representing the highest rate amongst treatment options in the survey.

Eighty-four participants (40 per cent) reported other attempted treatments for their post-Covid-19 parosmia not mentioned in our study survey. A list of these additional treatments mentioned by respondents and not previously addressed by our survey can be found in Supplement B. Alpha-lipoic acid was most often reported among the other therapies not included in the survey (18 respondents; 8 per cent). Of these respondents, six (33 per cent) reported improvement, and two (11 per cent) reported maintained benefit. The first improvement with alpha-lipoic acid was seen in approximately 15 days of treatment, with a treatment duration of up to 1 month (39 per cent), 1–3 months (28 per cent), and more than 3 months for the remaining third.

### Medical history

Sixty-two respondents (30 per cent) reported having pre-existing sinus or nasal diseases, including rhinitis (36 respondents), a deviated septum (7 respondents) and chronic sinusitis (13 respondents). Fourteen respondents (6 per cent) declared having pre-existing neurological diseases, including chronic migraine, concussion and Bell's palsy. In addition, 115 study participants (55 per cent of respondents) reported having pre-existing conditions such as depression (66 respondents, 57 per cent), anxiety (36 respondents, 31 per cent) and fibromyalgia (7 respondents, 6 per cent). Thirty-six respondents (17 per cent) declared regularly smoking cigarettes (47 per cent), cannabis (31 per cent) and vaping (22 per cent).

## Discussion

Parosmia, which previously has been related to post-viral, neurodegenerative conditions and head trauma, increasingly has been linked to qualitative and quantitative olfactory dysfunction post-Covid-19 infection, with many patients experiencing long-lasting consequences.^18,19^ Our survey assessed multiple aspects of patients with post-Covid-19 parosmia, expressing the overall timing of symptoms, SARS-CoV-2 variant type prevalence and exploring subjective efficacy of trialled therapies, to support the characterisation of post-Covid-19 parosmia as a distinct clinical entity and to help fill the gap of this condition's presentation in the literature.

In our study, the predominance of females among respondents (86 per cent) was higher than previously reported (73.5–74 per cent).^20,21^ In contrast, Fuccillo *et al*. reported a lack of association between gender and the incidence of olfactory disorders post-Covid-19.^9^ Generally, women seem to be more likely to use social media and be members of support groups, which could create a selection bias in survey studies like this. Further studies are warranted to clarify whether females are at a higher risk of post-Covid-19 parosmia.

Only 20 per cent of the participants were vaccinated prior to SARS-CoV-2 infection. Interestingly, vaccination status at the time of Covid-19 infection appears to be a factor in the onset of parosmia, with a shorter time to onset in those participants who were vaccinated. According to a study by Herman *et al*., which involved 442 Covid-19 patients, those with at least 14 days of immunisation had a 70 per cent lower chance of developing olfactory dysfunction when infected; nonetheless, these authors did not mention the term ‘parosmia’ in their study.^13^ The pathophysiology explaining such a protective effect for post-Covid-19 parosmia remains elusive in a study design such as the present one, yet warrants further investigation.

Study participants appear to share common triggers for their parosmia, including onion, garlic, meat and coffee, among others. In a study by Rashid *et al*., the most common trigger stimuli for parosmia were most odours for 46 per cent of participants, followed by perfume (22 per cent), any odour (10 per cent), frying smell (10 per cent) and meat (10 per cent).^21^ Our study participants reported overall symptom severity of 6 (scale of 1–10), which highlights the effect of this condition on their health status. In previous studies on Covid-19-related parosmia, severe parosmia was reported by 18–65 per cent of the participants.^21,22^

Parosmia has been associated with poor quality of life.^20,21,23,24^ In our cohort, significant weight loss was reported by most respondents due to Covid-19-related parosmia. Additionally, declared suicidal thoughts, with one suicide attempt, reflect how parosmia's negative effect on quality of life extends beyond the reduced enjoyment of food and emphasises the urgency of finding an effective therapy for these patients.

Full recovery from post-Covid-19 parosmia was reported by only 3 per cent of study participants, which aligns with previous studies that have stated full recovery from Covid-19-related parosmia of 4.4–8.5 per cent at the time of reporting.^21,22^ Based on a mean recovery time of more than 14 months in our results, researchers should be mindful of the need for long-term follow up in study protocols involving this condition.

Different treatment modalities for post-Covid-19 olfactory dysfunction, which included over-the-counter supplements, intranasal steroids, olfactory training and, more recently, stellate ganglion block, have been reported in the literature.^14,20,21,24^ In our study, smell training, nasal steroid sprays and vitamin A drops were the most trialled treatments. However, despite its low report rate, participants who had a stellate ganglion block reported the highest percentage of improvement in the study (*n* = 15, 45 per cent), with seven of them (21 per cent) reporting a maintained benefit of that treatment.

Autonomic dysregulation has been implicated as a potential mechanism for Covid-19-related olfactory dysfunction.^15^ Dysautonomia could be induced by the autonomic nervous system's response or maladaptation to pro-inflammatory cytokines leading to excessive sympathetic nervous system activity, and a stellate ganglion block could terminate the increased sympathetic nervous system activity, as has been seen in post-traumatic stress disorder.^15,24^ The results of a phase II clinical trial investigating stellate ganglion block for this indication (for which enrolment was completed in December 2022) are still pending publication (identifier: NCT05445921).^25^ Given the very low risk of complications with ultrasound guidance^26^ and the potential immediate response to treatment, this intervention should be prioritised for further studies.

- Post-coronavirus disease 2019 (Covid-19) parosmia symptoms can significantly affect quality of life; thus, there is a need for effective treatment of post-Covid-19 parosmia
- Eighty per cent of respondents were not vaccinated at the time of Covid-19 infection; the vaccinated group reported earlier parosmia onset than the unvaccinated, with a mean difference of 36 days
- Respondents experienced smell and taste loss 3 days after the first Covid-19 symptoms, with olfactory recovery reported 4 weeks later, followed by parosmia onset at 12 weeks post infection
- Depression was the most cited item regarding how parosmia affected the quality of life
- The most commonly used treatment was smell training, followed by nasal corticosteroid spray and vitamin A drops
- Stellate ganglion blockade had the highest rate of self-reported improvement among trialled treatments

The present study has several limitations. Firstly, recruitment from patient support groups hosted on social media introduces selection bias. Membership of such groups likely is influenced by age, gender and other social demographics. In addition, members with persistent olfactory dysfunction who have recovered are more likely to have become inactive in the discussion group or have left the group. Secondly, additional responder bias is expected, which might result in members with successful treatment outcomes being more likely to respond. The survey includes treatments used outside of a clinical trial setting, with the associated risk of a placebo response effect, and relies on self-report (although psychophysical tests of parosmia are lacking). Finally, it is impossible to calculate an actual response rate for this study, as the number of members in each group does not necessarily represent the number of active group members or the number of views of the survey invitation post.

## Conclusion

The current study evaluated the demographics and trialled treatment for Covid-19-related parosmia. Most participants were females (86 per cent), with altered quality of life due to parosmia reported by more than half of the participants. Most respondents had not recovered from their parosmia when completing the survey; the significant effect reported on quality of life highlights that this is an essential topic for future research. Participants reported trialling a wide range of treatments, with low rates of self-reported improvement. The questionnaire results demonstrated that stellate ganglion blockade had the highest rate of self-reported improvement, warranting further research in a formal trial setting.

## Supplementary material

For supplementary material accompanying this paper visit https://doi.org/10.1017/S0022215123000713. click here to view supplementary material
