# Supplementary material for: Coronavirus disease 2019 related parosmia: an exploratory survey of demographics and treatment strategies
Source: J Laryngol Otol. 2023 May 17;137(11):1256–60. doi: 10.1017/S0022215123000713 (PMC10627779; doi:10.1017/S0022215123000713)
Supplement: Supplementary file 1 [file S0022215123000713sup.zip › S0022215123000713sup001.pdf]

# Parosmia Survey

Please complete the survey below.

Thank you!

---

Age

__________________________________

---

Sex

◯ Female
◯ Male
◯ Not reported

---

What is your Country?

__________________________________

---

City

__________________________________

---

When did you first test positive for COVID-19?

__________________________________
(dd-mm-yy)

---

Do you know the strain of SARS-CoV-2 you had?

◯ Wild type (Wuhan)
◯ Alpha
◯ Beta
◯ Delta
◯ Omicron
◯ Unknown

---

Did you receive COVID-19 vaccine?

◯ Yes
◯ No

---

Date of FIRST COVID-19 vaccine dose:

__________________________________

---

Which vaccine brand name did you take on your first dose?

◯ Pfizer-BioNTech Comirnaty
◯ Moderna Spikevax
◯ Janssen (Johnson & Johnson)
◯ Novavax Nuvaxovid
◯ AstraZeneca Vaxzevria
◯ Medicago Covifenz
◯ Other

---

Did you receive a second dose?

◯ Yes
◯ No

---

Date of SECOND COVID-19 vaccine dose:

If none, please skip this question.

__________________________________

---

Which vaccine brand name did you take on your second dose?

◯ Pfizer-BioNTech Comirnaty
◯ Moderna Spikevax
◯ Janssen (Johnson & Johnson)
◯ Novavax Nuvaxovid
◯ AstraZeneca Vaxzevria
◯ Medicago Covifenz
◯ Other

| | |
|---|---|
| Did you receive a third dose? | ◯ Yes<br>◯ No |
| Date of THIRD COVID-19 vaccine dose:<br><br>If none, please skip this question. | __________________________________ |
| Which vaccine brand name did you take on your third dose? | ◯ Pfizer-BioNTech Comirnaty<br>◯ Moderna Spikevax<br>◯ Janssen (Johnson & Johnson)<br>◯ Novavax Nuvaxovid<br>◯ AstraZeneca Vaxzevria<br>◯ Medicago Covifenz<br>◯ Other |
| Did you receive a fourth dose? | ◯ Yes<br>◯ No |
| Date of FOURTH COVID-19 vaccine dose:<br><br>If none, please skip this question. | __________________________________ |
| Which vaccine brand name did you take on your fourth dose? | ◯ Pfizer-BioNTech Comirnaty<br>◯ Moderna Spikevax<br>◯ Janssen (Johnson & Johnson)<br>◯ Novavax Nuvaxovid<br>◯ AstraZeneca Vaxzevria<br>◯ Medicago Covifenz<br>◯ Other |
| How was your sense of smell/taste before COVID-19? | ◯ Absent<br>◯ Decreased<br>◯ Normal |
| Have you experienced any smell/taste disturbance during/after COVID? | ◯ Yes<br>◯ No |
| Did you lose smell immediately with COVID-19? If not immediately, how soon after COVID symptoms in days? | __________________________________<br>(In days ) |
| When did smell start returning (weeks after COVID-19 diagnosis)? | __________________________________<br>(In weeks) |
| When did your parosmia start? | __________________________________ |
| Have you had any improvement in your parosmia? | ◯ 0% (no improvement)<br>◯ 25% improvement<br>◯ 50% improvement<br>◯ 75% improvement<br>◯ 100% improvement |
| How soon after your parosmia started did you notice improvement? (in weeks) | __________________________________<br>(In weeks) |

What are the worst triggers for your parosmia?

(List up to 5)

__________________________________
(List up to 5)

How has parosmia affected your quality of life? (For example, Reduced appetite/ weight loss/ depression)

__________________________________

**Concerning your parosmia, please state:**

| | Yes | No |
|---|---|---|
| Have you seen primary care for this? | ◯ | ◯ |
| Have you seen an ENT for this? | ◯ | ◯ |
| Have you required hospitalization? | ◯ | ◯ |

Please give us more detail about your hospitalization.

__________________________________

Have you lost weight because of your parosmia?

◯ Yes
◯ No

Approximately what percentage of body weight have you lost?

0% 50 100%

*(Place a mark on the scale above)*

On a scale of 1 to 10, please rate the severity of your parosmia today:

0 (no problem at all) 5 10 (worst imaginable)

*(Place a mark on the scale above)*

Have you had COVID a second time?

◯ Yes
◯ No

When did you have COVID for the second time (approximately)?

__________________________________

Did your parosmia change after your second COVID illness?

◯ Worse
◯ Same
◯ Better

**Although there are no proven treatments for parosmia, have you tried any of the following treatments?**

| | Yes | No |
|---|---|---|
| Nasal steroid spray | ◯ | ◯ |
| Nasal steroid irrigation | ◯ | ◯ |
| Oral steroid | ◯ | ◯ |
| Smell training | ◯ | ◯ |
| Vitamin A drops | ◯ | ◯ |
| Sodium citrate spray | ◯ | ◯ |
| Stellate Ganglion block | ◯ | ◯ |
| Colloidal Silver spray | ◯ | ◯ |
| Lion's Mane | ◯ | ◯ |
| Other: | ◯ | ◯ |

Did you feel any improvement in your parosmia with the nasal steroid spray?
◯ Yes
◯ No

How long did it take to see some improvement with nasal steroid spray? (in days)
0 60 120 +

*(Place a mark on the scale above)*

Did nasal steroid spray treatment create a maintained benefit for you?
◯ Yes ◯ No

How long was the duration of your treatment with nasal steroid spray?
◯ 1 week ◯ 2 weeks
◯ 3 weeks ◯ 4 weeks
◯ 5 weeks ◯ 6 weeks
◯ 7 weeks ◯ 8 weeks
◯ 9 weeks ◯ 10 weeks
◯ 11 weeks ◯ 12 weeks
◯ over 12 weeks

Did you feel any improvement in your parosmia with the nasal steroid irrigation?
◯ Yes
◯ No

How long did it take to see some improvement with nasal steroid irrigation? (in days)
0 60 120 +

*(Place a mark on the scale above)*

Did nasal steroid irrigation treatment create a maintained benefit for you?
◯ Yes ◯ No

| | |
|---|---|
| How long was the duration of your treatment with nasal steroid irrigation? | ◯ 1 week ◯ 2 weeks<br>◯ 3 weeks ◯ 4 weeks<br>◯ 5 weeks ◯ 6 weeks<br>◯ 7 weeks ◯ 8 weeks<br>◯ 9 weeks ◯ 10 weeks<br>◯ 11 weeks ◯ 12 weeks<br>◯ over 12 weeks |
| Did you feel any improvement in your parosmia with the oral steroid? | ◯ Yes<br>◯ No |
| How long did it take to see some improvement with oral steroid? (in days) | 0 60 120 +<br>*(Place a mark on the scale above)* |
| Did oral steroid treatment create a maintained benefit for you? | ◯ Yes ◯ No |
| How long was the duration of your treatment with oral steroid? | ◯ 1 week ◯ 2 weeks<br>◯ 3 weeks ◯ 4 weeks<br>◯ 5 weeks ◯ 6 weeks<br>◯ 7 weeks ◯ 8 weeks<br>◯ 9 weeks ◯ 10 weeks<br>◯ 11 weeks ◯ 12 weeks<br>◯ over 12 weeks |
| Did you feel any improvement in your parosmia with the Smell training? | ◯ Yes<br>◯ No |
| How long did it take to see some improvement with the Smell training? (in days) | 0 60 120 +<br>*(Place a mark on the scale above)* |
| Did Smell training treatment create a maintained benefit for you? | ◯ Yes ◯ No |
| How long was the duration of your treatment with Smell training? | ◯ 1 week ◯ 2 weeks<br>◯ 3 weeks ◯ 4 weeks<br>◯ 5 weeks ◯ 6 weeks<br>◯ 7 weeks ◯ 8 weeks<br>◯ 9 weeks ◯ 10 weeks<br>◯ 11 weeks ◯ 12 weeks<br>◯ over 12 weeks |
| Did you feel any improvement in your parosmia with Vitamin A drops? | ◯ Yes<br>◯ No |
| How long did it take to see some improvement with Vitamin A drops? (in days) | 0 60 120 +<br>*(Place a mark on the scale above)* |
| Did Vitamin A drops treatment create a maintained benefit for you? | ◯ Yes ◯ No |

| | |
|---|---|
| How long was the duration of your treatment with vitamin A drops? | ◯ 1 week ◯ 2 weeks<br>◯ 3 weeks ◯ 4 weeks<br>◯ 5 weeks ◯ 6 weeks<br>◯ 7 weeks ◯ 8 weeks<br>◯ 9 weeks ◯ 10 weeks<br>◯ 11 weeks ◯ 12 weeks<br>◯ over 12 weeks |
| Did you feel any improvement in your parosmia with Sodium citrate spray? | ◯ Yes<br>◯ No |
| How long did it take to see some improvement with Sodium citrate spray? (in days) | 0 60 120 +<br>*(Place a mark on the scale above)* |
| Did Sodium citrate spray treatment create a maintained benefit for you? | ◯ Yes ◯ No |
| How long was the duration of your treatment with Sodium citrate spray? | ◯ 1 week ◯ 2 weeks<br>◯ 3 weeks ◯ 4 weeks<br>◯ 5 weeks ◯ 6 weeks<br>◯ 7 weeks ◯ 8 weeks<br>◯ 9 weeks ◯ 10 weeks<br>◯ 11 weeks ◯ 12 weeks<br>◯ over 12 weeks |
| Did you feel any improvement in your parosmia with Stellate Ganglion block? | ◯ Yes<br>◯ No |
| Did Stellate Ganglion block treatment create a maintained benefit for you? | ◯ Yes ◯ No |
| How long was the duration of your treatment with Stellate Ganglion block? | ◯ 1 week ◯ 2 weeks<br>◯ 3 weeks ◯ 4 weeks<br>◯ 5 weeks ◯ 6 weeks<br>◯ 7 weeks ◯ 8 weeks<br>◯ 9 weeks ◯ 10 weeks<br>◯ 11 weeks ◯ 12 weeks<br>◯ over 12 weeks |
| Did you feel any improvement in your parosmia with Colloidal Silver spray? | ◯ Yes<br>◯ No |
| How long did it take to see some improvement with Colloidal Silver spray? (in days) | 0 60 120 +<br>*(Place a mark on the scale above)* |
| Did Colloidal Silver spray treatment create a maintained benefit for you? | ◯ Yes ◯ No |

| | |
|---|---|
| How long was the duration of your treatment with Colloidal Silver spray? | ◯ 1 week ◯ 2 weeks<br>◯ 3 weeks ◯ 4 weeks<br>◯ 5 weeks ◯ 6 weeks<br>◯ 7 weeks ◯ 8 weeks<br>◯ 9 weeks ◯ 10 weeks<br>◯ 11 weeks ◯ 12 weeks<br>◯ over 12 weeks |
| Did you feel any improvement in your parosmia with Lion's Mane? | ◯ Yes<br>◯ No |
| How long did it take to see some improvement with Lion's Mane? (in days) | 0 60 120 +<br>*(Place a mark on the scale above)* |
| Did Lion's Mane treatment create a maintained benefit for you? | ◯ Yes ◯ No |
| How long was the duration of your treatment with Lion's Mane? | ◯ 1 week ◯ 2 weeks<br>◯ 3 weeks ◯ 4 weeks<br>◯ 5 weeks ◯ 6 weeks<br>◯ 7 weeks ◯ 8 weeks<br>◯ 9 weeks ◯ 10 weeks<br>◯ 11 weeks ◯ 12 weeks<br>◯ over 12 weeks |
| Other: Which Treatment? | __________________________________ |
| Other: Did you feel any improvement in your parosmia with [which_treatment]? | ◯ Yes<br>◯ No |
| Other: How long did it take to see some improvement with [which_treatment]? (in days) | 0 60 120 +<br>*(Place a mark on the scale above)* |
| Other: Did [which_treatment] create a maintained benefit for you? | ◯ Yes ◯ No |
| Other: How long was the duration of [which_treatment]? | ◯ 1 week ◯ 2 weeks<br>◯ 3 weeks ◯ 4 weeks<br>◯ 5 weeks ◯ 6 weeks<br>◯ 7 weeks ◯ 8 weeks<br>◯ 9 weeks ◯ 10 weeks<br>◯ 11 weeks ◯ 12 weeks<br>◯ over 12 weeks |
| What is your estimated total money expense in trying to treat your parosmia? (converted to USD) | __________________________________<br>(in US dollars ) |
| Do you have any pre-existing Sinus or nasal diseases (for example, Sinusitis, allergies, etc)?<br><br>If yes, please specify. ______ | ◯ Yes<br>◯ No |

| | |
|---|---|
| Do you have other conditions, such as depression, anxiety, or fibromyalgia?<br><br>If yes, please specify.<br><br>______ | ◯ Yes<br>◯ No |
| Do you have any pre-existing neurological diseases such as head trauma, multiple sclerosis, etc?<br><br>If yes, please specify!<br><br>______ | ◯ Yes<br>◯ No |
| Do you take any medications regularly?<br><br>If yes, please specify.<br><br>______ | ◯ Yes<br>◯ No |
| Do you regularly smoke cigarettes/vapes/Hookah/cannabis?<br><br>If yes, please specify.<br><br>______ | ◯ Yes<br>◯ No |
| Please add in any additional comments or thoughts you may wish to share regarding your parosmia: | __________________________________ |

Thank you very much for your time!
