# Supplementary material for: Coronavirus disease 2019 related parosmia: an exploratory survey of demographics and treatment strategies
Source: J Laryngol Otol. 2023 May 17;137(11):1256–60. doi: 10.1017/S0022215123000713 (PMC10627779; doi:10.1017/S0022215123000713)
Supplement: Supplementary file 1 [file S0022215123000713sup.zip › S0022215123000713sup002.docx]

Supplement B List of reported trialled treatments for post COVID parosmia that were not present within the study survey options.

| Trialled treatments Count (%) | |
| --- | --- |
| Alpha Lipoic Acid | 18 (8) |
| Turmeric drops | 4 (2) |
| Acupuncture | 6 (3) |
| Antibiotics | 2 (1) |
| Epigallocatechin gallate | 2 (1) |
| Zinc | 5 (2) |
| Melatonin | 1 (0.5) |
| Duloxetine | 1 (0.5) |
| Deep Transcranial Magnetic Stimulation | 1 (0.5) |
| Modafinil | 1 (0.5) |
| Chiropractic adjustment (atlas) | 1 (0.5) |
| Red light therapy | 4 (2) |
| CBD oil | 3 (1) |
| Vagus nerve stimulation | 3 (1) |
| Food desensitization – taste training | 4 (2) |
| Hair growth gummies containing Biotin | 1 (0.5) |
| Boiron - Sepia - 30C | 1 (0.5) |
| Thieves oil | 1 (0.5) |
| Ivermectin | 1 (0.5) |
| Pea-Lut | 1 (0.5) |
| Essential oils | 1 (0.5) |
| Nasal Rinse | 2 (1) |
| Adrenal Supplement | 2 (1) |
| Vitamin B12 | 1 (0.5) |
| Nebulized diluted food-grade peroxide | 3 (1) |
| Transcutaneous Electrical Nerve Stimulation (TENS) | 1 (0.5) |
| Iodine patch | 1 (0.5) |
| Sphenopalatine Ganglion Block | 1 (0.5) |
